# Surveillance of intestinal schistosomiasis during control: a comparison of four diagnostic tests across five Ugandan primary schools in the Lake Albert region

**DOI:** 10.1017/S003118201800029X

**Published:** 2018-03-21

**Authors:** Hajri Al-Shehri, Artemis Koukounari, Michelle C. Stanton, Moses Adriko, Moses Arinaitwe, Aaron Atuhaire, Narcis B. Kabatereine, J. Russell Stothard

**Affiliations:** 1 Department of Parasitology, Liverpool School of Tropical Medicine, Liverpool, L3 5QA, UK; 2 Ministry of Health, Asir District, Kingdom of Saudi Arabia; 3 Department of Clinical Sciences, Liverpool School of Tropical Medicine, Liverpool, L3 5QA, UK; 4 Lancaster Medical School, Lancaster University, Lancaster, LA1 4YG, UK; 5 Vector Control Division, Ministry of Health, P.O. Box 1661, Kampala, Uganda

**Keywords:** DNA-TaqMan^®^, Kato-Katz, latent class analysis, *Schistosoma mansoni*, SEA-ELISA, urine-CCA

## Abstract

Programmatic surveillance of intestinal schistosomiasis during control can typically use four diagnostic tests, either singularly or in combination, but these have yet to be cross-compared directly. Our study assembled a complete diagnostic dataset, inclusive of infection intensities, from 258 children from five Ugandan primary schools. The schools were purposely selected as typical of the endemic landscape near Lake Albert and reflective of high- and low-transmission settings. Overall prevalence was: 44.1% (95% CI 38.0–50.2) by microscopy of duplicate Kato-Katz smears from two consecutive stools, 56.9% (95% CI 50.8–63.0) by urine-circulating cathodic antigen (CCA) dipstick, 67.4% (95% CI 61.6–73.1) by DNA-TaqMan^®^ and 75.1% (95% CI 69.8–80.4) by soluble egg antigen enzyme-linked immunosorbent assay (SEA-ELISA). A cross-comparison of diagnostic sensitivities, specificities, positive and negative predictive values was undertaken, inclusive of a latent class analysis (LCA) with a LCA-model estimate of prevalence by each school. The latter ranged from 9.6% to 100.0%, and prevalence by school for each diagnostic test followed a static ascending order or monotonic series of Kato-Katz, urine-CCA dipstick, DNA-TaqMan^®^ and SEA-ELISA. We confirm that Kato-Katz remains a satisfactory diagnostic standalone in high-transmission settings but in low-transmission settings should be augmented or replaced by urine-CCA dipsticks. DNA-TaqMan^®^ appears suitable in both endemic settings though is only implementable if resources permit. In low-transmission settings, SEA-ELISA remains the method of choice to evidence an absence infection. We discuss the pros and cons of each method concluding that future surveillance of intestinal schistosomiasis would benefit from a flexible, context-specific approach both in choice and application of each diagnostic method, rather than a single one-size fits all approach.

## Introduction

Developing appropriate diagnostics tools, methods and protocols to track parasitic diseases before, during and after control is an important component within the multi-disciplinarity of parasitology. It has been previously highlighted (Stothard and Adams, 2014) and with regard to schistosomiasis, intestinal schistosomiasis poses a considerable public health burden in Uganda (Loewenberg, 2014). Since 2003 there has been an active national control programme against it (Kabatereine *et al.* 2006, 2007; Fenwick *et al.* 2009; Stanton *et al.* 2017), as primarily based on preventive chemotherapy campaigns (Montresor *et al.* 2012; Stothard *et al.* 2013). Despite much progress in the delivery of praziquantel (PZQ) treatments to school-aged children, infections with *Schistosoma mansoni* continue to be pervasive, particularly along the immediate shoreline of Lake Albert (Al-Shehri *et al.* 2016). Moving some 10–20 km inland, however, infection prevalence by school can decline dramatically, at least if measured by faecal egg-patency for if more sensitive diagnostic tools were used, such as urine-antigen dipsticks, such declines are less precipitous (Stothard *et al*. 2006, 2017*b*).

The incongruence between ‘estimated’ and ‘true’ prevalence is a well-known diagnostic dilemma in surveillance of intestinal schistosomiasis largely due to an operational compromise between imperfect detection tools and insufficient specimen sampling (Bergquist *et al.* 2009, 2015; Stothard *et al.* 2014; Utzinger *et al.* 2015; Weerakoon *et al.* 2015). Nonetheless, if control programmes are to be monitored effectively and also permit evidence-based adaptation or revision of control tactics (Tchuente *et al.* 2017), infection dynamics at an individual level need to be captured alongside any broader changes in the epidemiological landscape amenable to measurement (Hawkins *et al.* 2016; Stothard *et al.* 2017*a*). As the strive towards elimination grows (Hawkins *et al.* 2016; Colley *et al.* 2017), previous diagnostic shortcomings are revealed highlighting new diagnostic needs that guide future target product profiles (Utzinger *et al.* 2015; Weerakoon *et al.* 2015; Hawkins *et al.* 2016; Savioli *et al.* 2017; Tchuente *et al.* 2017).

At an individual level, often the school-aged child, the diagnostic repertoire for surveillance of intestinal schistosomiasis within national control programmes has remained surprisingly meagre; for many years it has been exclusively founded on parasitological methods alone (Stothard *et al.* 2014), with only sporadic application of serological methods (Chernet *et al.* 2017; Hinz *et al.* 2017). With the growing need for modernization and interest in adoption of more sensitive disease diagnostics in general (Mabey *et al.* 2004; Solomon *et al.* 2012; Stothard and Adams, 2014), in recent years there have been two important developments that centre upon scale-up in the use of urine-circulating cathodic antigen (CCA) dipsticks (Colley *et al.* 2013; Sousa-Figueiredo *et al.* 2013; Foo *et al.* 2015; Danso-Appiah *et al.* 2016; Greter *et al.* 2016; Kittur *et al.* 2016) and development of DNA-detection platforms with real-time PCR with parasite-specific TaqMan^®^ hydrolysis probes (ten Hove *et al.* 2008; Mejia *et al.* 2013; Easton *et al.* 2016). Furthermore, recent application of more sophisticated statistical modelling such as latent class analysis (LCA) (Hadgu *et al.* 2005), has advanced diagnostic tool performance comparisons beyond the direct need of a fixed reference ‘gold’ standard which, for schistosomiasis, is something we currently do not have (Shane *et al.* 2011; Ibironke *et al.* 2012; Koukounari *et al.* 2013; Beltrame *et al.* 2017).

In this study, we attempt to make a diagnostic comparison for surveillance of intestinal schistosomiasis in school children across five primary schools using four methods namely: microscopy of duplicate Kato-Katz smears from two consecutive stools, urine-CCA dipsticks, real-time PCR of stool with a *Schistosoma*-specific Taqman^®^ probe and serological analysis of finger-prick blood for antibodies against schistosome soluble egg antigen (SEA). Diagnostic congruence was first assessed by empirical cross-tabulations, assuming a ‘gold standard’, then later by LCA with disease prevalence by school also estimated with a LCA model.

## Methods

### Study area, participants and ethical approval

**Fig. 1.**
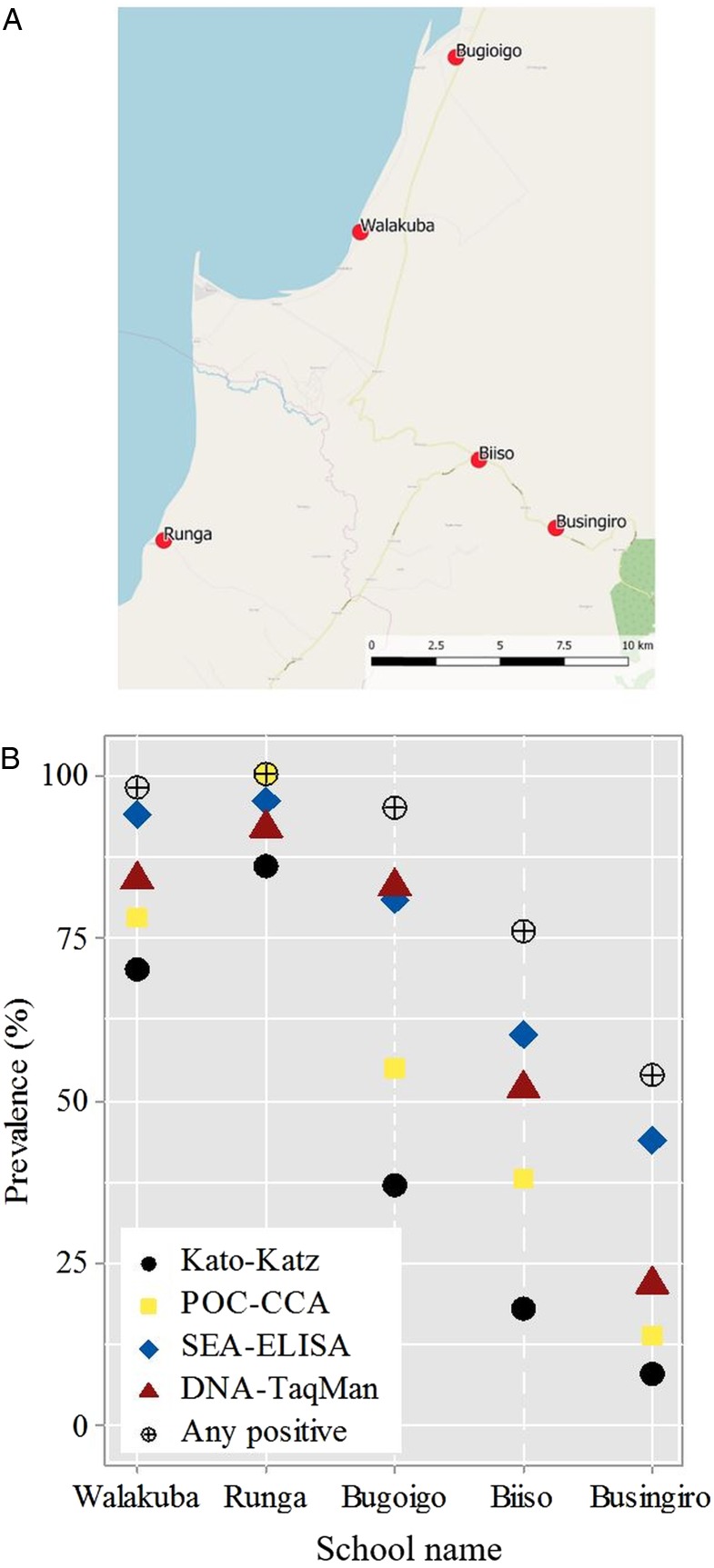
(A) Schematic map of the five sampled primary schools in the Lake Albert region, the blue area indicates Lake Albert. (B) Estimated prevalence of *Schistosoma mansoni* by school for each examined diagnostic test; prevalence by any positive test criterion is also illustrated.

Field sampling and examinations of children took place in May 2015 in five primary schools in Buliisa District located within the Lake Albert region, three of which have been visited previously as sentinel surveillance sites of the national control programme (Kabatereine *et al.* 2007) and the global positioning system locations (GPS) known (Fig. 1). The schools Walakuba (GPS 01°50.323N, 031°22.740E), Bugoigo (GPS 01°54.004N, 031°24.750E) and Runga (GPS 01°43.828N, 031°18.603E) were located on the immediate shoreline while Biiso (GPS 01°45.516N, 031°25.236E) and Busingiro (GPS 01°44.090N, 031°26.855E) were over 10 km away inland which aimed to represent the current control landscape across high- and low-endemic settings, respectively.

After obtaining written informed consent and verbal assent, a pre-target of 60 children of balanced gender aged between 5 and 10 years of age were enrolled and requested to provide two stool samples on consecutive days, a single urine sample and single finger-prick blood sample. Children were also interviewed with a standardized questionnaire to ascertain recent PZQ treatment history. All participants were provided with a single PZQ (40 mg/kg) treatment by the attending nurse following WHO guidelines (Montresor *et al.* 1998). The Ugandan Council for Science and Technology and the Liverpool School of Tropical Medicine granted approval for this study.

### Diagnostics: faecal microscopy with Kato-Katz

Duplicate Kato-Katz thick smear slides (41.7 mg templates) were prepared from each stool received after first sieving through a 212 *µ*m metal mesh (Montresor *et al.* 1998). Schistosome eggs were viewed by microscopy (×100 magnification), quantified and expressed as eggs per gram (EPG) of faeces with the intensity of infection classified as: light (1*–*99 EPG), medium (100*–*399 EPG) and heavy (⩾400 EPG) following the WHO guidelines (Montresor *et al.* 1998). For later DNA analysis, a 0.8 g aliquot of sieved stool was each prepared and stored in 95% ethanol before transportation to the UK for processing.

### Diagnostics: schistosome urine-antigen CCA dipsticks

The commercially available urine-CCA dipstick was used to test for schistosome antigens in each urine sample received following manufacturer's instructions (Rapid Medical Diagnostics, Pretoria, South Africa). The test result was classified by visual inspection against a colour chart as used previously (Sousa-Figueiredo *et al.* 2013), by two individuals as negative, trace (±), light positive (+), medium positive (++) and heavy positive (+++). In this setting, all trace reactions were later considered to be positive as justified previously upon biological causality and by prior epidemiological analyses (Standley *et al.* 2010*a*, *b*; Sousa-Figueiredo *et al.* 2013; Adriko *et al.* 2014).

### Diagnostics: schistosome serology with *SEA*-enzyme-linked immunosorbent assay (ELISA)

Finger-prick blood was taken from each child and antibodies for SEA were tested using 1 : 40 dilution of harvested sera using a field-based ELISA test following manufacturer's instructions (IVD Inc.; Carlsbad, USA). Upon completion, the micro-titre plate was placed on a white card to view the visual colour of each reaction as graded into pale yellow (light positive), yellow (medium positive) and dark yellow (heavy positive) as recorded previously (Stothard *et al*. 2009).

### DNA diagnostics: TaqMan^®^ real-time PCR

After transfer to the UK, each aliquot of stool was spiked with Phocine Herpes Virus (PhHV-1) to act as an internal control for each DNA extraction and later real-time PCR assay for inhibition following protocols of Meurs *et al.* (2015) which targetted a 77 base pair segment within the ribosomal internal transcribed spacer (ITS-2) region which can be identified using *S. mansoni* (GenBank: AF503487) as reference sequence (Obeng *et al.* 2008; Meurs *et al.* 2015). Schistosome DNA was detected with the *Schistosoma*-specific primers of Ssp48F (GGT CTA GAT GAC TTG ATY GAG ATG CT) and Ssp124R (TCC CGA GCG YGT ATA ATG TCA TTA) and TaqMan^®^ probe Ssp78T (ROX – TGG GTT GTG CTC GAG TCG TGGC – Black Hole Quencher 3) as developed by (Obeng *et al.* 2008; Meurs *et al.* 2015). DNA-TaqMan^®^ assays were performed in a Chromo-4 with Opticon monitor Version 3.1. (Biorad, Hemel Hempstead, UK) with Biorad iQ™ supermix and thermal cycling conditions of 15 min at 95 °C, followed by 45 cycles, each of 15 s at 95 °C and 60 s at 60 °C. The infection intensity was classified according to *C*_t_ values: negative (*C*_t_ > 45), light positive (35 > *C*_t_ ⩽ 45), medium positive (25 > *C*_t_ ⩽ 35), and heavy positive (*C*_t_ ⩽ 25).

### Data management and statistical analysis

All data collected in the field and processed in the laboratory were recorded on proforma data sheets. These were then double entered in Microsoft Excel prior to the generation of summary tables for prevalence and intensity of infection (Tables 1 and 2). Empirical estimates of sensitivity, specificity, negative predictive value and positive predictive value was calculated in R statistical package v 2·10·1 (The R Foundation for Statistical Computing, Vienna, Austria) and SPSS software (v 24.0, SPSS Inc., IBM, USA) assuming the urine-CCA as the ‘gold’ standard against the remaining three diagnostic tests (Table 3). For percentage values, 95% confidence intervals (95% CI) were estimated using the exact method (Armitage and Berry, 1994). We have decided to assume the urine-CCA as the gold standard for our descriptive analyses (i.e. empirical estimates of diagnostic performance), since there have been extensive evaluations of urine-CCA dipsticks (Colley *et al.* 2013) and WHO recommendation of its use in surveillance mapping (Danso-Appiah *et al.* 2016).

**Table 1.** Prevalence (%) of *Schistosoma mansoni* according to each diagnostic test across five primary schools with 95% confidence intervals

	School name					
	Walakuba (*n* = 50)	Runga (*n* = 50)	Bugoigo (*n* = 58)	Biiso (*n* = 50)	Busingiro (*n* = 50)	Total (*N* = 258)
Diagnostic method	% (95% CI)	% (95% CI)	% (95% CI)	% (95% CI)	% (95% CI)	% (95% CI)
Kato-Katz	70.0 (56.8–83.1)	86.0 (76.0–95.9)	37.9 (25.0–50.8)	20.0 (8.5–31.4)	8.0 (0.2–15.7)	44.1 (38.0–50.2)
Urine-CCA dipstick	78.0 (66.1–89.8)	100 (NA)	55.1 (41.9–68.3)	38.0 (24.0–51.9)	14.0 (4.0–23.9)	56.7 (50.8–63.0)
SEA-ELISA	94.0 (87.1–100.8)	96.0 (90.3–101.6)	81.0 (70.6–91.4)	60.0 (45.9–74.0)	44.0 (29.7–58.2)	75.1 (69.8–80.4)
DNA-TaqMan	84.0 (73.4–94.5)	92.0 (84.2–99.7)	82.7 (72.7–92.7)	52.0 (37.6–66.3)	24.0 (10.1–33.8)	67.4 (61.6–73.1)
Positive by any test	98.0 (93.9–102.0)	100 (NA)	94.8 (88.9–100.0)	76.0 (63.7–88.2)	54.0 (39.6–68.3)	84.8 (80.4–89.2)

**Table 2.** Intensity of infection categories for *Schistosoma mansoni* by each examined diagnostic test across the five primary schools

Diagnostic test and intensity category	School name					
	Walakuba (*n* = 50)	Runga (*n* = 50)	Bugoigo (*n* = 58)	Biiso (*n* = 50)	Busingiro (*n* = 50)	Total (*N* = 258)
	(%) *n*	(%) *n*	(%) *n*	(%) *n*	(%) *n*	*N* (%)
Kato-Katz^a^						
Negative	15 (30.0)	7 (14.0)	36 (60.1)	40 (80.0)	46 (92.0)	144 (55.8)
Light (<100 EPG)	5 (10.0)	7 (14.0)	16 (27.6)	3 (6.0)	1 (2.0)	32 (12.4)
Medium (100–399 EPG)	10 (20.0)	5 (10.0)	3 (5.2)	5 (10.0)	2 (4.0)	25 (9.7)
Heavy (≥400 EPG)	20 (40.0)	31 (62.0)	3 (5.2)	2 (4.0)	1 (2.0)	57 (22.1)
Urine-CCA						
Negative	11 (22.0)	0 (0.0)	26 (44.8)	31 (62.0)	43 (86.0)	111 (43.0)
Light (+, incl. trace)	5 (10.0)	10 (22.0)	19 (32.8)	7 (14.0)	4 (8.0)	45 (17.4)
Medium (++)	6 (12.0)	9 (18.0)	7 (12.1)	6 (12.0)	1 (2.0)	29 (11.2)
Heavy (+++)	28 (56.0)	31 (62.0)	6 (10.3)	6 (12.0)	2 (4.0)	73 (28.3)
SEA-ELISA						
Negative	3 (6.0)	2 (4.0)	11 (19.0)	20 (40.0)	28 (56.0)	64 (24.8)
Light (+, incl. trace)	4 (8.0)	3 (6.0)	14 (24.1)	16 (32.0)	13 (26.0)	50 (19.3)
Medium (++)	33 (66.0)	19 (38.0)	27 (46.5)	11 (22.0)	8 (16.0)	98 (37.9)
Heavy (+++)	10 (20.0)	26 (52.0)	6 (10.3)	3 (6.0)	1 (2.0)	46 (17.8)
DNA TaqMan^®^						
Negative (*C*_t_ > 45)	8 (16.0)	4 (8.0)	10 (17.2)	24 (48.0)	38 (76.0)	84 (32.5)
Light (35 > *C*_t_ ⩽ 45)	4 (8.0)	2 (4.0)	20 (34.5	18 (36.0)	8 (16.0)	52 (20.1)
Medium (25 > *C*_t_ ⩽ 35)	19 (38.0)	9 (18.0)	19 (32.8)	5 (10.0)	2 (4.0)	54 (20.9)
Heavy (*C*_t_ ⩽ 25)	19 (38.0)	35 (70.0)	9 (15.5)	3 (6.0)	2 (4.0)	68 (26.3)

^a^Duplicate faecal smears from two consecutive stools.

**Table 3.** Empirical estimates of sensitivity (SS), specificity (SP), negative predictive value (NPV) and positive predictive value (PPV), Cohen's kappa for each diagnostic test against urine-CCA dipstick as ‘gold standard’

Evaluating diagnostic test	Urine-CCA as reference ‘gold standard’						Cohen's kappa (95% Cls)
	Negative (%)	Positive (%)		Measurement estimate % (95% Cls)		Diagnostic accuracy % (95% Cls)	
DNA-TaqMan^®^			Total (%)				
Negative	62 (24.0)	22 (8.5)	84 (32.6)	Sensitivity	85.0 (78.4–89.9)	72.5 (66.7–77.6)	0.4 (0.3–0.5)
Positive	49 (18.9)	125 (48.5)	174 (67.4)	Specificity	55.9 (46.6–64.7)		
Total (%)	111 (43.0)	147 (57.0)	258 (100.0)	PPV	71.8 (64.7–77.9)		
				NPV	73.8 (63.5–82.0)		
SEA-ELISA							
Negative	59 (22.9)	5 (1.9)	64 (24.8)	Sensitivity	96.6 (92.3–98.5)	77.9 (72.5–82.5)	0.5 (0.4–0.6)
Positive	52 (20.2)	142 (55.0)	194 (75.2)	Specificity	53.2 (43.9–62.2)		
Total (%)	111 (43.0)	147 (57.0)	258 (100.0)	PPV	73.2 (66.6–78.9)		
				NPV	92.2 (82.9–96.6)		
Kato-Katz							
Negative	110 (42.6)	34 (13.2)	144 (55.8)	Sensitivity	76.9 (69.2–82.9)	86.4 (81.7–90.1)	0.73 (0.6–0.9)
Positive	1 (0.4)	113 (43.8)	144 (44.2)	Specificity	99.1 (95.1–99.8)		
Total (%)	111 (43.0)	147 (56.9)	258 (100.0)	PPV	99.1 (95.2–99.8)		
				NPV	76.4 (68.8–82.6)		

Subsequently, to tackle the inherent problems with diagnostic measurement error, we employed a LCA and full information maximum likelihood estimation (Table 4). LCA allows grouping of categorical data (in the current study not infected and infected from the diagnostic tests under examination) into latent classes indicating *S. mansoni* infection *via* a probability model. Given the well-known epidemiological landscape of Lake Albert region, such a model was designed to allow LCA estimated prevalence of *S. mansoni* to vary by school (Table 4). Through this approach, model-based estimates of sensitivity and specificity across diagnostic tests without assuming a gold standard were also obtained.

**Table 4.** Latent class analysis (LCA) estimates of sensitivity and specificity and LCA model of prevalence of *Schistosoma mansoni* by school with 95% CIs for each diagnostic method

Diagnostic method	Sensitivity	Specificity
Kato-Katz	84.4% (76.0–92.9)	100% (NA)
Urine-CCA	99.1% (97.3–100)	89.3% (80.9–97.6)
SEA-ELISA	97.7% (95.1–100)	49.5% (39.4–59.6)
DNA-TaqMan^®^	90.2% (84.2–96.2)	57.5% (48.6–66.5)
School name	Prevalence by LCA model	
Walakuba	75.7% (62.9–88.5)	
Runga	100.0% (NA)	
Bugoigo	49.7% (35.0–64.5)	
Biiso	27.0% (12.2–41.9)	
Busingiro	9.6% (9.0–18.4)	

The classification certainty of this model was evaluated through entropy; values of entropy near one indicate high certainty in classification while values near zero indicate low certainty (Celeux and Soromenho, 1996). LCA assumes the relationships between the observed variables (i.e. diagnostic tests in the current study) are accounted for by their class membership and thus conditioning on class membership (i.e. the disease status in the current study) such that if the model estimated disease status is misclassified by one test, the probability that it will be misclassified by another test will not be affected. We assessed this assumption by speculating the standardized residuals for each response pattern from the diagnostic tests as estimated from the LCA model. Further technical details of these models in the context of schistosomiasis have been described elsewhere and thus they are not repeated here (Ibironke *et al.* 2012). The LCA model was fitted using MPlus version 7.3 (Muthén and Muthén, 1998–2012).

## Results

### Prevalence of intestinal schistosomiasis

A total data set was assembled from 258 children with a prevalence of intestinal schistosomiasis by each diagnostic test presented, see Table 1. Overall prevalence of intestinal schistosomiasis was: 44.1% (95% CI 38.0–50.2) by microscopy of duplicate Kato-Katz smears from two consecutive stools, 56.9% (95% CI 50.8–63.0) by urine-CCA dipstick, 67.4% (95% CI 61.6–73.1) by DNA-TaqMan^®^ and 75.1% (95% CI 69.8–80.4) by SEA-ELISA.

The prevalence of infection at Runga and Walakuba was observed to be highest, exceeding 50% in all diagnostic tests, whereas prevalence of infection at Busingiro was lowest falling well short of 50% by any test, although pooling infection status upon being positive by any test revealed that just under half of the children attending this school could be considered ‘free’ from infection. For the total dataset, just over a quarter of children (*n* = 69) could be considered to have no evidence of intestinal schistosomiasis.

The geographical proximity of each of the five schools to Lake Albert shoreline is depicted in schematic in Fig. 1A; on-the-ground shortest distance to the lake shoreline can be ranked in the following order of Walakuba (0.2 km), Runga (0.4 km), Bugoigo (0.9 km), Biiso (9.4 km) and Busingiro (13.2 km). Notably, both Runga and Bugoigo schools are located for safety and convenience on slightly higher ground behind each village so as not to flood, which during wetter periods has detrimentally affected Walakuba in the past (J.R.S., personal observation). Whilst diagnostic comparisons are made on the basis of binary data, it is worth noting that infection intensity also varied by school setting, in that ‘heavy intensity’ infections or ‘strong positive’ by any test were particularly common at Runga but were rare at Busingiro, Table 2. As shown in Fig. 1B the changing prevalence by school for each method is clearly visible in that the prevalence of inferred from each diagnostic test typically followed a static ascending order or monotonic series of Kato-Katz, urine-CCA dipstick, DNA-TaqMan^®^ and SEA-ELISA, although the relative position of the estimated prevalence by urine-CCA at Runga slightly exceeds SEA-ELISA and DNA-TaqMan^®^.

### Empirical and LCA modelling of estimates of diagnostic performance

Assuming the urine-CCA as an arbitrary gold standard, the diagnostic performance for the three remaining tests is shown along with diagnostic accuracy and Cohen's kappa statistic, Table 3. The sensitivity of SEA-ELISA is the highest (96.6%) but also has the lowest specificity (53.2%), with the highest negative predictive value of all methods. By contrast, the sensitivity of Kato-Katz is the lowest (76.9%) but also has the highest specificity (99.1%), with the highest positive predictive value of all methods.

On the basis of LCA analysis the sensitivity and specificity of each method can be estimated on the basis of their latent class assignment which highlights the trade-off between diagnostic specificity (i.e. false positive) and sensitivity (i.e. false negative). In this analysis, sensitivity and specificity of SEA-ELISA and urine-CCA are broadly equivalent with DNA-TaqMan^®^ appearing to have slightly lower sensitivity and specificity. Estimating the prevalence of infection by school with LCA, Table 4, reveals a lower prevalence than that on the basis of positivity by any test but follows the same static ascending order or monotonic series (Fig. 1B). It is evident that at Runga intestinal schistosomiasis is universal whereas at Busingiro around 9.6% of children are suspected of harbouring infections.

The LCA model generated similar sensitivity for SEA-ELISA and urine-CCA but was slightly lower for DNA-TaqMan^®^, Table 4. Kato-Katz was again shown through LCA to have the highest specificity among all the four tests. The specificity of 89.3% (95% CI 80.9–97.6) for the urine-CCA test was acceptable but for the SEA-ELISA and the DNA-TaqMan, specificities were less so and estimated to be 49.5% (95% CI 39.4–59.6) and 57.5% (95% CI 48.6–66.5), trending as with empirical calculations, see Table 3. Furthermore, LCA estimated infection prevalence of *S. mansoni* by the school to be lower than that on the basis of positivity by any test (for the latter see Fig. 1B). Nevertheless, both of these approaches suggested that intestinal schistosomiasis was universal at Runga, however, at Busingiro LCA estimated a prevalence of *S. mansoni* infection to be 9.6% (95% CI 9.0−18.4), much lower than that revealed by positivity upon any test. Finally, the entropy of the LCA model was estimated to be 0.921. This indicated a clear delineation of classes in the fitted model standardized residuals for each response pattern from the four diagnostic tests from this model were between −2 and 2, evidencing that local independence of the four diagnostic tests is not obviously violated.

## Discussion

Owing to the complicated developmental and population biology of the schistosome within the mammalian host, it is well known that accurate detection of intestinal schistosomiasis by any biomarker can be problematic and has been the topic of at-length discussions previously (Bergquist *et al.* 2009, 2015; Stothard *et al.* 2014; Utzinger *et al.* 2015). Foremost, the insensitivity of the Kato-Katz, especially in the detection of light egg-patent infections or in patients with a recent history of PZQ treatment, is perhaps the most obvious obstacle to overcome (Kongs *et al.* 2001; Koukounari *et al.* 2013; Leuenberger *et al.* 2016).

Indeed, how we debate and assess the significance of egg-negative infections is changing alongside measuring morbidity associated with intestinal schistosomiasis which goes beyond what Kato-Katz assessments can offer (King, 2015). Nevertheless, Kato-Katz can still be promoted as a field-applicable standalone and appropriate in high-endemic settings, as seen here in both Runga and Walakuba, where prevalence and intensity of infection were high. Nonetheless, Kato-Katz has several deficits when applied to lower transmission settings, as exemplified by the other schools sampled here and is more misleading perhaps than informative. To compensate, de Vlas *et al.* (1993) developed a useful corrective prevalence chart which took into account infection intensity; however, its uptake was not as good as anticipated (de Vlas *et al.* 1993). It is also outside the scope of the present paper to discuss economic cost–benefit of faecal microscopy (Meheus *et al.* 2015) other than that mobile microscopy with handheld devices offers some attractive cost-saving solutions for surveillance of intestinal schistosomiasis in high-endemic areas (Stothard *et al.* 2005; Bogoch *et al.* 2014). However, as control programmes move forward towards elimination, the Kato-Katz methodology will be inappropriate and will be unable to provide sufficient quality epidemiological information for precision mapping of disease foci (Tchuente *et al.* 2017). The latter is pivotal in the local intensification of delivery of treatments and surveillance interventions to confirm interruption of transmission (Rollinson *et al.* 2013; Stothard *et al.* 2017*a*). Indeed from the information reported here, we would suggest that control efforts in locations such as Busingiro should be intensified rather than reduced.

Of the remaining diagnostic methods, the diagnostic pros and cons of each method have been discussed elsewhere often using the ASSURED framework (Bergquist *et al.* 2009; Stothard *et al.* 2014; Utzinger *et al.* 2015). DNA-TaqMan^®^ methods are, however, increasingly gaining favour and offer a multiplex DNA-platform for co-detection of several neglected tropical diseases as well as many other infectious agents (Verweij and Stensvold, 2014); much more so than other any other current biomarker method can provide (ten Hove *et al.* 2008; Solomon *et al.* 2012; Mejia *et al.* 2013; Easton *et al.* 2016). There also is the suggestion that DNA-TaqMan^®^ could become an acceptable ‘gold’ standard (Meurs *et al.* 2015), and whilst we ultimately share some enthusiasm in this there are some impediments to discuss. Foremost, DNA-TaqMan^®^ requires specialist equipment and is not currently amenable to point-of-contact settings although there is growing interest in the use of more field-friendly methods (Minetti *et al.* 2016), such as loop-mediated iso-thermal amplification (LAMP) (He *et al.* 2016) and recombinase polymerase amplification (RPA) (Rosser *et al.* 2015). Nonetheless, from our results here the DNA-TaqMan^®^ has been somewhat outperformed upon consideration of Table 4. In our opinion, perhaps the most significant advantage of DNA-based platform is that DNA-TaqMan^®^ assays can cross-over into environmental monitoring through detection of environmental (e)DNA and therefore broaden the vision of schistosomiasis control in general potentially uniting clinical and environmental surveillance (Rollinson *et al.* 2013; Stothard *et al.* 2017*a*).

In the absence of a ‘gold’ standard diagnostic test and complexity of the changing epidemiological landscape in which tests are being applied in Uganda (Standley *et al.* 2010*a*, *b*; Adriko *et al.* 2014; Al-Shehri *et al.* 2016), our analysis presented in Table 3 postulated that urine-CCA dipsticks could be an ‘error-free’ standard which, in Table 4, was further explored by LCA. Here the probabilistic statistical model applied does not assume any ‘gold’ standard and therefore points towards the urine-CCA as having near-optimal diagnostic scores of sensitivity (99.1%) and specificity of (89.3%). Moreover, these scores are significantly better than those reported previously by empirical comparisons (Stothard *et al.* 2006) and illustrate how advances in statistical modelling developed elsewhere on urine-CCA dipsticks (Knopp *et al.* 2015; Koukounari *et al.* 2013) can provide a deeper insight into diagnostic score evaluations over and above simple empirical calculations (Colley *et al.* 2013; Danso-Appiah *et al.* 2016). Nonetheless a theoretical issue of adopting LCA-models exclusively is an assumption of independence of tests which, given the biological biomarkers employed here could be somewhat confounded; Kato-Katz detects eggs directly, DNA-TaqMan^®^ measures *Schistosoma*-DNA in stool (presumably from excreted eggs) and SEA-ELISA detects antibodies to secreted egg-antigens, thus these three methods are somewhat interrelated to similar biomarkers of the egg itself although will have each have differing physical, biochemical and physiological components. However, the urine-CCA dipstick is less directly connected to egg-biomarkers for it utilizes carbohydrate-antigens released from feeding worms of either sex and hence offers an alternative biomarker appraisal. Since violations of the conditional independence assumption can lead to biased LCA estimates of accuracy and prevalence, performing and reporting checks of whether assumptions are met is essential which was why we compared LCA estimates of diagnostic performance with empirical ones, drawing conclusions for each of the diagnostic tests used. In addition, speculation of standardized residuals from the LCA model indicated that the assumption of local independence of the four diagnostic tests under examination was not obviously violated.

Over and above the routine diagnostic scores of sensitivity, specificity, negative and positive predictive values with or without LCA models, however, it is also necessary to further consider each diagnostic tool against the ASSURED criteria. This seeks to understand whether a diagnostic test can be used at scale and is ultimately useful in several clinical and epidemiological surveillance settings (Mabey *et al.* 2004; Peeling *et al.* 2006; Stothard and Adams, 2014). The roll-out of the urine-CCA test has been discussed previously (Stothard, 2009) and it is pleasing to see it become further endorsed at the policy level (Danso-Appiah *et al.* 2016). The most desirable features of this test are its affordability, stable commercial production, the use of urine-sampling, the speed of test and a short time to obtain results which has a very pragmatic consideration for the end-user in this emphasis. All of the above potentially make dissemination of epidemiological results back to the local community obtained by the urine-CCA dipstick much quicker, which is vital to increase local ownership of preventive chemotherapy campaigns in future (Tchuente *et al.* 2017).

## Concluding remarks

The study has shown that intestinal schistosomiasis continues to be a public health challenge on the shoreline of Lake Albert which now presents as a heterogenous epidemiological landscape of high- and low-transmission settings. A total of four diagnostic tests were each assessed regarding contemporary surveillance for intestinal schistosomiasis finding that Kato-Katz sampling is a satisfactory diagnostic standalone in high-transmission settings but in low-transmission settings should be augmented or replaced by urine-CCA dipsticks. DNA-TaqMan^®^ appears suitable in both endemic settings though is only implementable if resources permit. In low-transmission settings, SEA-ELISA remains the method of choice to evidence an absence of infection. In the dearth of a diagnostic ‘gold’ standard for intestinal schistosomiasis, LCA offered useful computations of diagnostic performance between tests.
